# Editorial

**Published:** 1862-05

**Authors:** 


### Editorial.

EDITORIAL CORRESPONDENCE.—CONGESTIVE OR PERNICIOUS FEVER.—The following letter from Dr. ORMSBY, in relation to a case of sudden death will be read with interest; and we copy it entire, for the purpose of bringing the subject of pernicious fever more fully before our readers. The letter is as follows:—

### CASE OF CEREBRAL CONGESTION.

On the morning of 28th March, 1862, was called to see J. C. of the 18th Regt. Ill. Vols., American, farmer, aged 22 years, of rather short stature, but stout, and usually very healthy. I found him laboring under an attack, as I thought, of remittent fever, presenting the ordinary symptoms, with nothing to indicate that it was of a severe grade or an unusual type. I found the face flushed, the eyes partially suffused, pupils natural, tongue soft and moist—with only a light coat, the pulse 100, full, and of ordinary strength.

The bowels were somewhat relaxed, and complaint was made of pain in the head and bones; was informed he had some fever during the previous day, which had subsided in the night. His prescription was, pulv. Dover, hydrarg. cum creta, aa grs. xxx, M., divide in five parts; one to be taken every two hours, with mucilage of gum arabic, until the subsidence of the fever, when he was to have: quinine grs. xx; Dover grs. xv; pulv. capsic. grs. v, divided into five equal parts, and taken in the same manner as the former. Next morning, found him apparently considerably better; but still complaining of pain in the head and bones. Fever had not entirely subsided, but was much lower, and I gave the nurse directions to give the tonic at once, so that he should get the twenty grains of quinine within the next ten hours. This, as I subsequently learned, was done, his fever not rising so high as on the day previous. I heard no more of the case until 7 o'clock A.M., the following day (March 30th), when I was sent for in haste to visit my patient. I found him insensible, lying on his back, with flushed face, dilated pupils, strong pulse—but not more than 80 to the minute, and upon raising his head found tonic spasm of the muscles of the back, amounting to slight opisthotonus. The hands and feet were warm, the skin on the extremities full, and the circulation to all appearance good. The breathing was a little slower than natural and stridulous: expiration being well performed, but inspiration accompanied by a sound similar to that heard in pertussis.

I immediately made a copious application of cold water to the head and neck; sinapisms to the abdomen, wrists, and ankles; and let fall two drops of ol. tiglii upon the tongue. Before we had completed these operations, I noticed that inspiration became more difficult, and the heart became much exerted. In a few minutes more the face assumed a purple hue; bloody foam in large quantities burst from the lips; and the man expired within a half hour of the time of my arrival. The great amount of blood which flowed from the mouth strongly suggested a broken vessel in the lungs: how this was I cannot say. The face was full of blood, and the entire body quite warm immediately after death. I should mention that a few minutes before death, the action of the heart was so strong and tumultuous as to be very plainly visible to the eye, and to shake the patient's entire body.

Upon inquiry, I learned that he had said, early in the morning, that he felt much better, and wanted some breakfast cooked and brought to him. He then got up, went out of the tent, stayed a few minutes, came back and lay down again on his bed. When his breakfast was brought, he said he felt worse, and would not eat. About 15 minutes afterward, I was called and found him in the condition described, which was so suddenly fatal. Now, I have taken the liberty to call this a case of congestion of the brain; and believe it to have been produced by miasmatic influence; but as to the precise pathological condition, I must own I am at fault. It is much to be regretted that unavoidable circumstances prevented a post mortem. But such being the case, let me enquire: What injury to the brain or spinal cord would produce such a defect in the respiratory apparatus? Sudden dropsy of the arachnoid, produced by paralysis of that membrane, has been suggested to me; but I cannot yet see my way clearly through the case upon that hypothesis, and so beg leave to sit back and object, until further explanation is made. I should be very much obliged to the Editor of the EXAMINER (or any other medical man of large experience,) for a minute statement of the inferred pathology of this case, and suggestions in the way of treatment. Such an exposition would be of very great interest to me, and perhaps to others, as I am informed that the case is isolated from others of a similar nature *only* by extraordinary severity.

O. B. ORMSBY, Assistant-Surgeon,
18th Regt. Ill. Volunteers.

The case thus fully described, undoubtedly belongs to that variety of *malarious* fevers variously designated by writers as *Congestive*, *Pernicious*, and *Malignant*. From an intimation in the above letter, we infer that attacks of the same variety, though less rapidly fatal, have been somewhat frequent among the volunteers on the Tennessee River, during the month of March. If we remember that the weather there, during that month, was much warmer than here, equally wet, and the soil highly favorable for the development of malaria, we shall have no difficulty in accounting for the prevalence of such cases, so far as they may depend on the presence of malaria. The following letter from Dr. NEYMAN, referring to another and distant section of the country, and giving a brief but interesting account of the symptoms of the same variety of fever, is of much interest in this connection. The writer calls it a "dreadful malady," prevailing at the date of his letter, May 16, 1862, but we regret that he does not tell us how *early* the first cases occurred. The letter is as follows:—

CEDAR BLUFFS, IOWA, *May* 16, 1862.

DEAR DOCTOR:—There is at present a dreadful malady prevailing in and about the Grinnell in this State, which has resisted the best directed efforts of our physicians. It has been noticed quite extensively in our newspaper literature, but I do not remember of seeing anything in the journals. I have not had an opportunity of investigating it fully; therefore, what I may say will be premature and unsatisfactory, and far from authoritative. I have conversed with several very intelligent physicians, whose opportunities for observation have been ample, and from what I have ascertained from them, and by personal observations, I have come to the following:—

*Symptoms.*—The premonatory symptoms which furnish an invariable indication of its approach are: for two or three evenings before the final paroxysm, the patient feels chilly; draws his coat tightly around him; draws near the fire, if there exists any. He thinks nothing of this, attributing it to a change in the atmosphere. Coincident with this are dull aching pains in the bones; lassitude; non-inclination to change positions; languor; &c. The final paroxysm—which almost invariably occurs in the evening, and almost as certainly proves fatal—consists of complete collapse; cold extremities, with but little chilliness; great apathy; recklessness of life; comatose; replies with difficulty to questions, and then sluggishly, relapses immediately into comatose condition. In fact, all the symptoms usual in what is known as congestion or "sinking" chill, only in a magnified degree.

*Treatment.*—If the premonitions are heeded, or the patient can be safely guided through the first *chill*, brandy and quinine proves as infallible a remedy as quinine for our ordinary intermittent fever, or sulphur for the scabies.

*Cause, Miasma.*—Our country had been flooded by rains and melted snow until our soil was completely saturated; the hot days coming dried up our land so suddenly that the previous mud became a resisting impervious crust. Is the disease anything else but an unusually malignant form of the *Congestive Chills?*

This account has been drawn up hastily. I thought it might possess some interest to you. I remain yours, respectfully,

E. H. NEYMAN.

The months of March and April, in this city and vicinity, were unusually cold and wet; and attacks of pneumonia and pleurisy were more frequent than in ordinary seasons.

Many of the cases of pneumonia were accompanied by plain indications of a malarious influence, while others speedily assumed a strongly typhoid character. During the latter part of February, several thousand prisoners were sent from Fort Donelson to Camp Douglas, located on the Southern margin of this city. Many of these were speedily attacked with pneumonia of a very severe grade. During all the month of March, the grounds of the camp continued very wet and the weather cold. It was not until the middle of April that the soil began to be comfortably dry and some of the days warm. From the middle of April to the 10th of May, pneumonia has been less frequent, both in the city and the camp; but both have furnished several cases of severe and rapidly fatal *pernicious fever* or "congestive chills." One of the physicians to the camp informed me, that during one week not less than ten or twelve cases of this disease occurred among the prisoners, and a large majority of them terminated fatally during the first or second paroxysm. About the same period of time, several cases of this variety of fever occurred in different parts of the city, but without any direct connection with the camp. And cases have continued to occur in the city, though few in number, until the present time. Most of the cases presented symptoms strictly corresponding with those detailed in the books, and briefly stated by Dr. NEYMAN; and yet there were some deviations of a striking and interesting character. For instance, among the first cases that came under my care, was that of Mr. M. a native of Ireland, living in the extreme South part of the city. He was naturally strong and healthy, but somewhat addicted to the use of alcoholic stimulants. When first called to see him, he was suffering from a peculiar and severe pain across the abdomen of a paroxysmal character, and steady pains in the back and limbs. He had been complaining three or four days, and on careful inquiry, I was so well satisfied that the disease was of malarious origin, that I at once prescribed powders, each containing sulphate of quinia, three grains; sulphate of morphia, one-third of a grain; calomel, two grains; to be given every two hours, until the abdominal pains were relieved, and subsequently every four hours, until six doses had been taken. The patient was speedily relieved, and for two or three days continued so much better that he called at my office,—full two miles from his residence. About the fourth day, he was seized with such violent and excruciating pains in the middle of the thighs, that I was again called for. I saw him in the evening, and in addition to the peculiar and indiscribable pain in both thighs, his whole cutaneous surface was cool, congested, dingy in color; the face pinched and haggard; the mind listless,—though restless and almost constantly changing position; and the pulse very small, weak, and frequent. Indeed, I was very forcibly struck with the resemblance of the symptoms to those resulting from the shock of a severe mechanical injury, as the crushing of a limb under the wheel of a railroad car. Yet he was in a sitting posture and appeared entirely rational. The remedies prescribed appeared to exert no influence whatever. The pulse grew weaker until it could no longer be felt, and the skin more livid and cold until death, which took place before morning.

Another case, which occurred only a few days since, was that of a very poor Irish woman, in the South-west part of the city. She had been complaining of feeling unwell and chilly for two days previous to any severe symptoms. Then a more decided chill occurred, accompanied by a great sense of exhaustion, lasting two or three hours. A partial reaction took place, accompanied by vomiting and purging, partly produced, no doubt, by some infusion of senna, given without any medical advice. It was after this had been going on nearly 18 hours, that I was called to see her. Found her with a very sunken and haggard expression of countenance; livid surface; cool extremities; pulse small, weak, and 140 per minute; mouth dry; constant thirst, but prompt vomiting of whatever was taken into the stomach; also frequent reddish serous discharges from the bowels, and an almost constant and severe pain in the umbilical region. The mind was listless and dull but entirely rational. I directed mustard sinapisms to the epigastrium and spine, and small but frequently repeated doses of morphine and soda, to arrest the vomiting and diarrhœa. Regarding the attack, however, as a case of irregular intermittent, aggravated by an injudicious use of a senna purge, I also prescribed a powder consisting of sulphate of quinine, 3 grs.; pulv. opium, 1 gr.; calomel 1 gr.; to be given every three hours. The next day, I found the patient remarkably improved. The abdominal pain, vomiting, and diarrhœa had entirely ceased, and the patient thought herself almost well. The color of the skin was not good, and the pulse remained soft and weak.

I warned the attendants that there was great danger of a renewal of dangerous symptoms during the next twenty-four hours, and enjoined the faithful exhibition of efficient anti-periodic doses of quinine, with nourishment for at least two days longer. Owing to stupidity and ignorance, these directions were not faithfully carried out, and about noon of the following day, I was again urgently requested to see her. About two hours elapsed before I could answer the call, and I then found her in the last struggles of death. On making inquiry, I learned that she had remained quite comfortable until about 10 o'clock A.M., when she was seized with a most excruciating pain in the middle of one thigh. This was directly followed by all the symptoms of extreme prostration, and death followed in about five hours. It will be seen that in both these cases the fatal paroxysm was ushered in by an anomalous and severe pain in the middle of the thighs. In another case, the attack was commenced by a violent pleuritic pain in the lower part of one side, followed by a rapid failure of respiration and death. In all the other cases coming under my observation, the symptoms were substantially the same as those described by Dr. NEYMAN.

The occurrence of cases of *pernicious* fever of the malarious type, during the months of March and April, in the interior of Tennessee, Iowa, and this city, would seem to indicate some general tendency to malarious fevers of a more malignant type than ordinary. Though the cases have not been very numerous in any one locality, yet occurring early in the season, the question arises whether they do not indicate a more extensive prevalence of the same class of fevers during the coming summer and autumn. That the case related by Dr. ORMSBY, and those alluded to by Dr. NEYMAN, as well as those which have occurred in this city, are genuine cases of *pernicious* fever or "sinking chills," I have no doubt. All those occurring in this city, and those in the army on the Tennessee River, were exposed to malaria or the ordinary cause of periodical fever, together with some of the more efficient causes of fever of a typhus type. To make my own views of the essential pathology of these highly dangerous cases understood, it is necessary to premise, that all the organized tissues of the human body are endowed with two elementary properties, viz.: *Susceptibility*, or a capacity to be acted upon or to receive impressions; and *vital affinity*, or that power by which the organic atoms of every structure are made to assume a certain relation to each other, and to attract from the blood some ingredients and reject others. These elementary properties are capable of being increased or diminished by various exterior influences. Some of these influences excite or exalt both these properties, disturbing in the same direction all the functions of the system, and causing what the old writers termed sthenic or synochal fever, and the moderns simple irritative or inflammatory fever. Other influences depress both properties, of course carrying the functions of the system in the same direction, and producing the typhus class of febrile diseases. Still another class of agents of a more specific character, are capable of increasing one of these properties while it diminishes the other, producing general disturbance or febrile phenomena of a peculiar and specific character, such as we see in the periodical and eruptive classes of fevers.

The primary action of malaria or the efficient cause of periodical fever, is such as to exalt the *susceptability* and diminish the *vital affinity* of the whole system. This is the first link in the chain of morbid action, from which arise the functional derangements and changes in the composition of the blood, that so constantly mark the progress of this class of diseases. If the cause acts with moderate intensity and uncomplicated with other causes, the result will be the production of *simple* intermittents. If the malaria acts in conjunction with other causes of such a nature as to determine a higher grade of excitement, the resulting fever will be of that variety which has been styled by Dr. DRAKE, inflammatory intermittents and remittents. On the other hand, if the circumstances of the season, locality, or individual are such as to give the malaria a more energetic effect on the vital affinity of the tissues, it may speedily so far suspend that affinity as to greatly retard all the organic or molecular changes in the tissues, suspending the production of animal heat, arresting secretion, impairing capillary circulation, and in an hour, so far enfeebling both circulation and respiration as to endanger an immediately fatal result.

But the circumstances co-operating with the action of the malaria may be such as to dangerously retard the vital affinity in only one organ or tissue, instead of the whole composing the body. Thus, in the case related by Dr. ORMSBY, the suspension of vital action or affinity took place in the brain and medulla oblongata, suspending mental functions and respiratory movements to a great extent, while the heart depending for nerve force on the ganglionic centres continued to act vigorously. This disparity between the respiratory and cardiac functions, doubtless led to the rapid apoplectic engorgement of the pulmonary tissue, and sanguineous effusion into the bronchial tubes furnishing the bloody mucus, that flowed so abundantly from the air passages at the time of death. If pernicious fever consists, as we have supposed, in a primary lesion of the elementary properties of one or all of the tissues, it is easy to see why quinine, which when given in doses of from three to five grains, powerfully increases vital affinity while it directly diminishes morbid susceptibility, proves an efficient and almost certain remedy if given in the formative stage, or before the patient is too deeply sunk in the fatal paroxysm. For these cases it should be given in full doses, and if combined with any other stimulant, we should prefer strong coffee, cayenne pepper, or camphor, to the brandy mentioned by Dr. NEYMAN. But the great difficulty in the treatment of these cases is, in the management of the paroxysm itself—in restoring sufficient play of vital affinity to enable the tissues to respond to the action of quinine or anything else. In a strongly marked case recently under my care, the paroxysm was broken and reaction was established under the use of the following:—

℞—Sulph. Quinine, ........................30 grs.
Aromat. Sulph. Acid, .................ʒjj,
Water, ..................................℥jj. Mixed.

I gave a teaspoonful in a tablespoonful of strong coffee every half-hour. Between each of these doses I gave a fluid drachm of a saturated solution of chlorate of potassa. Mustard sinapisms were also placed over the spine. After reaction was established, full anti-periodic doses of quinine, with two or three alterative doses of calomel, were given every three hours, until the patient was safe from a renewal of the paroxysm. If vomiting or diarrhœa existed, I would add to the above solution of quinine a small proportion of sulphate of morphia. The only additional remedy for establishing reaction in these dreaded paroxysms, in the efficacy of which we have any confidence, is the thorough application of the cold douche, followed by dry friction, or what is better—the alternate application of the cold wet sheet and the warm and dry blanket. But we have no space to pursue the subject further.

---

Uterine Hemorrhage.—Dr. H. Wanzer, of this city, sends us an account of a case of Uterine Hemorrhage, occurring in a lady at the 4th month of her term of nursing, and asks whether it was "an attempt of nature to re-establish the menstrual flow, or was it a case of early pregnancy?" From the fact that the hemorrhage came on gradually; was unaccompanied by either uterine pains or abdominal tenderness; continued without being apparently much influenced by internal remedies, about the usual time of the monthly flow, and then ceased under the influence of the tinct. ferri murias, we should regard it as a case of menorrhagia.

---

New York Ophthalmic Hospital.—The Directors of the above Institution having leased the spacious old mansion of Peter Cooper, Esq., at the corner of Fourth Ave. and 28th St., removed their Hospital to the above place, on the 1st inst. The location is a central one, being near the terminus of the Northern and Eastern Railways, and convenient to the poor from all parts of the city, besides being in the vicinity of the Medical Colleges, giving Students of Medicine an opportunity to witness operations and to attend the Ophthalmic cliniques at the Hospital. The attending Surgeons are Drs. Mark Stephenson, John P. Garrish, and Marcus P. Stephenson.

Consulting Surgeon, Professor Valentine Mott, M.D.

The building has been thoroughly repaired for the accommodation of patients. The price of board is $3,50 per week. Advice and medicine to the poor from all parts of the United States gratuitously given. That distinguished philanthropist of New York, Peter Cooper, is President of the Association. Over 10,000 patients have been attended since its organization in 1852.

---

General Literature and Art.—The Eclectic Magazine of Foreign Literature. W. H. Bidwell, editor and publisher. Published at No. 5 Beekman Street, New York.

The June number of this most excellent monthly of foreign literature is just received. In addition to the full complement of instructive, important, and entertaining matter usually found in this magazine, it contains a beautifully engraved portrait of His Royal Highness, Prince Albert, the late Prince Consort of Queen Victoria.

---

"Illinois Farmer," and "Prairie Farmer."—We receive regularly and promptly both these interesting and valuable agricultural Journals. The first is published in Springfield, Ill., and the second in this city. They certainly deserve a very wide circulation throughout the great agricultural community of the North-west.

